# TYRAY-Functionalized Alginate Bioinks for 3D Bioprinting Support Stem Cell Culture and Endothelial Network Formation

**DOI:** 10.1021/acsbiomaterials.5c01132

**Published:** 2025-11-20

**Authors:** Vaclav Chochola, Karolina Spustova, Josef Lavicky, Anna Golunova, Jakub Pospisil, Jana Dvořáková, Ilya Kotelnikov, Mario Kandra, Libor Streit, Ariel Szklanny, Shulamit Levenberg, Vladimir Proks, Ales Hampl, Josef Jaros

**Affiliations:** † Department of Histology and Embryology, Faculty of Medicine, 37748 Masaryk University, Kamenice 5, Brno 62500, Czech Republic; ‡ International Clinical Research Center, St. Anne’s University Hospital Brno, Pekarska 53, Brno 656 91, Czech Republic; § 86879 Institute of Macromolecular Chemistry, Czech Academy of Sciences, Heyrovskeho nam. 2, Prague 16200, Czech Republic; ∥ Department of Burns and Plastic Surgery, University Hospital Brno & Faculty of Medicine, Masaryk University, Jihlavska 20, Brno 62500, Czech Republic; ⊥ Department of Plastic and Aesthetic Surgery, St. Anne’s University Hospital & Faculty of Medicine, Masaryk University, Pekarska 53, Brno 656 91, Czech Republic; # Department of Biomedical Engineering, 26747 Technion − Israel Institute of Technology, Haifa 32000, Israel; ¶ Department of Molecular Cell Biology, Institute for Cancer Research, Oslo University Hospital, 0379 Oslo, Norway

**Keywords:** alginate, bioink, peptide functionalization, TYRAY, 3D bioprinting, pluripotent stem cells, spheroid fusion, endothelial networks, vascularization

## Abstract

3D bioprinting is transforming tissue engineering by enabling spatial arrangement of cells and cell aggregates within supportive hydrogels. Among available materials, alginate remains widely used for its biocompatibility, printability, and cost-effectiveness. However, its bioinert nature and lack of adhesive moieties restrict its capacity to support essential processes like cell adhesion, migration, and proliferation. In this study, we propose a comprehensive approach to enhance alginate hydrogels focusing on stem, stromal, and endothelial cell types to support extended growth and vascular network formation. Key innovations include the incorporation of the TYRAY peptide in 3D alginate hydrogels—its first application in this context—to promote cell adhesion and migration, accompanied by Ca­(OH)_2_-modified surfaces for stable hydrogel anchoring and an ultrasonic mist cross-linking to preserve 3D structure fidelity. Functionalization with the TYRAY peptide significantly enhanced cell proliferation, promoted multicellular spheroid fusion, and supported endothelial network development in comparative culture setting. Together, these findings establish this defined, xeno-free alginate system as a versatile bioink material suitable for 3D culture and bioprinting applications.

## Graphical abstract

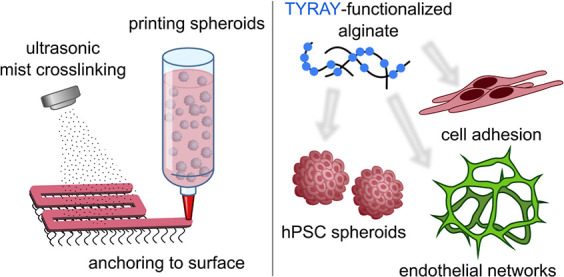

## 1 Introduction

The growing need for fabricated tissues as alternatives to organ transplantation has driven advancements in regenerative medicine.^1^ Human pluripotent stem cells (hPSCs) possess the remarkable ability to differentiate into diverse cell types, providing a promising pathway for the creation of functional tissues.^2–5^ Their potential to be used individually or as self-organized spheroids and organoids as building blocks for larger tissue models has unlocked new possibilities, particularly when combined with 3D bioprinting technologies, which extend traditional 3D culture by enabling spatially controlled deposition of bioinks, cells, and multicellular aggregates.

3D bioprinting enables the fabrication of structures with high shape, dimensional, and stratification accuracy.^6,7^ However, its effectiveness heavily depends on the properties of the bioinks, i.e., materials such as biocompatible polymers, biomolecules, and living cells, used independently or in combinations. The choice of bioink plays an essential role in the printed structure integrity and quality, with the focus on the optimal biological and mechanical support tailored to specific cell type, partly determined by its natural or synthetic origin.^8–10^

Alginate is widely used in 3D bioprinting due to its biocompatibility, tunable mechanical properties, and FDA (Food and Drug Administration) approval.^11–15^ However, its inert nature limits direct cell adhesion and interaction and in 3D cultures often leads to the formation of isolated 3D cell colonies rather than interconnected multicellular structures.^16^ To overcome this, alginate-based bioinks are typically modified with two complementary strategies, either by combining with additional polymers or by functionalizing alginate with bioactive peptides to support cell adhesion and migration.

First, introducing an additional polymer can enhance printability, but also porosity, addressing the issue of cell isolation and providing space for the cells to grow within the alginate.^16–20^ In extrusion bioprinting, gelatin is frequently incorporated as a sacrificial material and rheological modifier, enhancing the fidelity of bioprinted constructs. However, due to its lack of covalent binding with ionically stabilized alginate, gelatin is progressively leached out during culture.^16^

Second, long-term cell interaction with alginate bioinks relies on appropriate modifications, which are particularly critical for hPSCs. These cells require a microenvironment that promotes cell–cell interactions, adhesion, and nutrient exchange while simultaneously addressing the challenges associated with their poor survival as single cells and high sensitivity to microenvironmental cues.^21^ Extracellular matrix (ECM) proteins such as fibronectin, laminin, and vitronectin are often used to promote cell adhesion, but their undefined composition and limited compatibility with alginate restrict their application in defined bioinks.

Integrin-mediated adhesion is a central mechanism through which stem and endothelial cells interact with their microenvironment. Human pluripotent stem cells predominantly express αvβ5 and α6β1 integrins,^22,23^ supporting attachment and survival on vitronectin- and laminin-based substrates. Endothelial cells preferentially engage αvβ3 and α5β1 integrins, which regulate adhesion, migration, and angiogenic signaling.^24,25^ Notably, αvβ5 and αvβ3 integrins are also expressed in multiple hPSC-derived lineages—including endodermal, endothelial, and hematopoietic cells—where they contribute to differentiation and morphogenesis.^26–28^ Rather than attempting to target all integrin subtypes, we sought a ligand that would be broadly compatible with hPSCs, their derivatives, and endothelial cells, ensuring functional applicability across tissue types.

Peptide functionalization has emerged as an effective strategy to introduce integrin-binding sites in 3D culture and bioprinting systems. Commonly utilized adhesion motifs include fibronectin-derived RGD, which supports adhesion of mesenchymal and many somatic cell types^29–33^ yet remains insufficient for hPSCs.^34,35^ Endothelial-selective REDV promotes angiogenesis,^36^ while laminin-derived YIGSR or IKVAV motifs support neural, endothelial, and epithelial cultures.^37–39^ More advanced systems combined multiple ligands, such as cyclic RGD with AG73 to engage both integrins and syndecans^40^ or RGD/BFP-1/QK peptide formulations that synergistically enhance osteogenesis, angiogenesis,^41^ and proangiogenic factor secretion.^42^ Collectively, these studies highlight that peptide efficacy depends not only on ligand identity but also on density, clustering, and spatial distribution within the alginate matrix. While multipeptide systems show promise, they require complex chemistries and often display cell-type selectivity, limiting their universality.

In this context, we selected the bone sialoprotein-derived GGGNGEPRGDTYRAY (TYRAY) peptide, which uniquely engages both αvβ5 and αvβ3 integrins. This dual specificity is particularly relevant for hPSCs (αvβ5) and endothelial cells (αvβ3)^43,44^ while remaining compatible with MSCs, whose integrin expression is more variable.^23,45–48^ Compared with laminin- or vitronectin-derived sequences, TYRAY offers a single integrin-targeted motif capable of supporting multiple clinically relevant cell types. Prior studies confirmed its efficacy for hPSC adhesion and self-renewal under 2D conditions,^35,48,49^ but its application in 3D hydrogels has not been explored. This knowledge gap provided the rationale and novelty of this study.

Here, we hypothesize that incorporating the TYRAY peptide into the alginate hydrogel can enhance cell adhesion, promote migration, and facilitate the expansion of various stem and endothelial cell types relevant to tissue engineering. Moreover, this strategy could provide a defined, xeno-free alternative to natural matrices such as Matrigel.

We have previously shown that thanks to advanced techniques such as alginate amidation and Cu-catalyzed azide–alkyne cycloaddition, peptide-rich material ensuring a favorable microenvironment for stem cell growth can be prepared.^38^ Building on this foundation, the present study integrates biochemical and engineering innovations to improve the performance of alginate-based bioinks for 3D bioprinting. Specifically, we introduce the TYRAY-functionalized alginate as a bioactive, xeno-free matrix capable of supporting diverse cell types—including hPSCs, ADSCs, and endothelial cells—while maintaining structural integrity and biological functionality over extended culture.

In parallel, we optimized several key technical parameters—surface anchoring via Ca­(OH)_2_ modification, gentle CaCl_2_ mist cross-linking, and incorporation of multicellular spheroids—to enhance construct stability and reproducibility. The bioink formulation and printability were optimized using acellular printing tests, whereas its biological performance was validated in 3D dome and spheroid-based cultures, providing comprehensive evaluation prior to full bioprinted applications. Together, these advances address key limitations of conventional alginate bioinks and enable the formation of viable, interconnected multicellular structures and endothelial networks, advancing the development of physiologically relevant 3D tissue models.

## 2 Methods

### 2.1 Preparation of Unmodified Stock Polymer Solutions

Alginates were prepared as 5% (w/v) stock solution. Alginic acid sodium salt (180947, Sigma-Aldrich) was dispersed in DMEM/F-12 (21331020, Gibco) and dissolved while shaking at 70 °C under sterile conditions for 48–72 h. The solution was centrifuged for 5 min at 10,000*g*, and the supernatant was transferred into sterile tubes. As this solution still contained speckles and impurities, an optically clear alginate solution was prepared by dissolution of sodium alginate powder in deionized water to 0.5% (w/v) under vigorous stirring at 50 °C overnight instead. The solution was then filtered through 0.45 or 0.22 μm filter, frozen, and lyophilized. The final product was dissolved in DMEM/F-12 overnight to form a 5% stock solution. Gelatin (G1890, Sigma-Aldrich) was dissolved in DMEM/F-12 at 37 °C to produce a stock 20% (w/v) solution.

### 2.2 Sterilization of Polymer Solutions

Sterilization of alginate materials was performed by ^60^Co gamma irradiation using a laboratory irradiator (OG-LK0632-01, VF a.s., Czech Republic). Alginate was exposed to 200 Gy for 2 h at 24 °C, either as a powder/lyophilizate (“gamma dry”) or as a 5% alginate stock solution (“gamma wet”). Other tested approaches included exposure to UV-C light for 30 min in a biosafety cabinet, in both powder (“UV dry”) and reconstituted forms (“UV wet”), respectively, or by autoclaving dissolved stock 5% solution at 121 °C for 10 min (Tuttnauer 3870ELV). CELLINK A-RGD (IK2000110301, CELLINK, Sweden) was supplied as 3% sterile solution. Gelatin was sterilized using the “gamma dry” method. Afterward, all manipulations with polymer powder and solutions were done strictly under sterile conditions. Sterility of solutions was verified experimentally by placing 100 μL of the respective stock solution into 1 mL of DMEM/F-12 without antibiotics and incubated in cell culture incubator for a week. The potential microbial growth was monitored daily via visual examination under the microscope and changes in medium pH.

### 2.3 Preparation of Working Solutions

Working solutions of all alginates (unmodified, TYRAY, CELLINK A-RGD) were prepared by dilution of stock solutions in DMEM/F-12 to final concentrations of 2.5% alginate (A2.5), 2.5% alginate with 10% gelatin (A2.5G10), and 1% alginate with 10% gelatin (A1G10). Pre-cross-linked A2.5 alginate was prepared by adding 1 M CaCl_2_ to the medium before mixing with 5% stock alginate. Final concentration in pre-cross-linked A2.5 was 10 mM CaCl_2_.

### 2.4 Comparison of Rheological Properties

Relative rheological properties were compared by placing a 75 μL drop of 5% alginate solutions on clean glass. The glass was tilted vertically for 10 s, and the sliding distance of each droplet was measured. The distance was normalized to the sliding distance of the glycerol drop (14550, PENTA, Czech Republic). The method was selected as a simple, reproducible, semiquantitative comparison of flow characteristics without the need for special instrumentation, as inspired by other studies.^50,51^ Addition of a reference sample (glycerol) and retaining the same 90 degree angle and measurement interval ensure higher accuracy of the method, compared to the simple observation of solution flow in inverted tubes.^52–54^

### 2.5 Synthesis of the TYRAY Peptide

Azidoacetic-GGGNGEPRGDTYRAY-NH_2_ (N_3_-TYRAY) was synthesized by the standard Fmoc/tBu solid-phase method on a TentaGel Rink Amide R resin (0.18 mmol NH_2_/g). An automatic microwave peptide synthesizer (Liberty Blue, CEM Corporation, Matthews, NC, USA) with default DIIC/OxymaPure coupling and piperidine deprotection cycles was used. The peptide was cleaved from the resin using a CF_3_COOH/thioanisole/triisopropylsilane/H_2_O mixture (95/3/1/1, v/v/v/v) and precipitated in diethyl ether. The crude product was purified by preparative reversed-phase HPLC (Kinetex 5 μm C18 100 Å, 250 × 21.2 mm column) and obtained at >95% purity. The identity of the peptide was confirmed by MALDI-TOF mass spectrometry.

### 2.6 Modification of Alginate with the TYRAY Peptide

Bioactive peptide conjugation to alginate was made according to the previously published two-step protocol.^49^ At the first step, alginic acid sodium salt was functionalized with propargylamine. For that, 0.1 M alginic acid sodium salt (180947, Sigma-Aldrich, Germany) was dissolved in reverse osmosis (RO) water overnight. Solutions of 0.01 M propargylamine and 0.1 M 4-(4,6-dimethoxy-1,3,5-triazin-2-yl)-4-methylmorpholinium chloride (DMTMM; both Sigma-Aldrich, Germany) in water were prepared right before the reaction. Solutions were mixed and left to react for 24 h under ambient conditions with stirring. Afterward, the reaction mixture was dialyzed and lyophilized. The structure was confirmed by NMR analysis. In the second step, modified alginate was conjugated to the bioactive TYRAY peptide via Cu­(I)-catalyzed azide–alkyne cycloaddition. The click reaction between a water solution of 0.0025 M TYRAY (purged with nitrogen) and modified 0.025 M alginate took place. The reaction was catalyzed with the system of 0.0005 M ascorbic acid (Sigma-Aldrich, Germany) and the 0.0005 M Cu–THPTA complex prepared from copper­(II) sulfate pentahydrate (CuSO_4_) (Lach-Ner, Czech Republic) and tris­(3-hydroxypropyltriazolylmethyl)­amine (Sigma-Aldrich, Germany). The reaction mixture was left to react for 24 h under ambient conditions with stirring and later dialyzed against RO water, lyophilized, and used for subsequent analysis. For further experiments, the material was treated the same as unmodified alginate (sterilized by 200 Gy of gamma irradiation and reconstituted to 5% stock solution).

### 2.7 Peptide Conjugation with the Use of EDC/NHS

For the lower degree of substitution, the standard EDC/NHS conjugation method was used. Sodium alginate (0.05 M) was dissolved in MES buffer (pH 6.5) overnight. Solutions of the peptide (TYRAY, 0.006 M), EDC (0.06 M), and NHS (0.12 M) in MES buffer were prepared right before the reaction. The final concentration of sodium alginate and the peptide in the reaction mixture was 2% (w/v). The reaction mixture was left to react for 24 h under ambient conditions with stirring and later dialyzed against RO water, lyophilized, and used for subsequent analysis.

The substitution degree (DS) was calculated from the NMR spectral analysis (Figure S4) as a ratio between the peak integrals assigned to the tyramine signal (*I*_tyr_) in the peptide sequence and the hydrogen signal at C1 of the guluronic acid (*I*_alg_)­

1 DS=ItyrNH2.56×Ialg×100%

Obtained materials had DS 8 mol % (0.375 mmol/g of alginate) and 1.8 mol % (0.089 mmol/g of alginate) and were denoted as TYRAY-8DS and TYRAY-1.8DS, respectively.

### 2.8 Ionic Cross-Linking of Alginate

The initial cross-linking of alginate bioinks was done with a 100 mM CaCl_2_ solution (16770-31000, PENTA, Czech Republic). To minimize disturbances to the shape and surface integrity of the gel, three different techniques were applied. (1) Pipetting—A CaCl_2_ solution was gently pipetted on the top of alginate in the culture dish. (2) Mechanical spraying—Structures were cross-linked with spraying of CaCl_2_ solution from a distance of 20 cm using a piston spray. (3) Ultrasonic mist—A fine mist of CaCl_2_ solution was generated using a custom-made ultrasonic humidifier with a 16 mm, 108 kHz transducer (sourced from AliExpress, Figure S1G). The mist was applied from a distance of 5–10 cm to establish a gentle, nondisruptive surface set that preserved feature fidelity. Constructs were then submerged in 100 mM CaCl_2_ for 30–60 s and subsequently cultured in medium supplemented with 5 mM CaCl_2_ medium, allowing Ca^2+^ to diffuse and equilibrate through the gel during early culture. Thus, this step prioritized surface integrity rather than instantaneous full-depth cross-linking.

### 2.9 Ca­(OH)_2_ Modification of Tissue Culture Plastic

To anchor alginate hydrogels onto the cell culture surfaces, cell culture plates (TPP) were treated with oxygen plasma (Plasma Etch PE-75) for 5 min and immediately covered with a saturated (0.16%, w/v) solution of Ca­(OH)_2_ (102047, Supelco). The solution was aspirated, and plates were dried at room temperature (RT) and sterilized under UV for 30 min. Plates were stored at +4 °C.

### 2.10 Cell Culture

As lines of pluripotent stem cells, we employed human embryonic stem cell (hESC) cell line CCTL 14 (hPSCreg name: MUNIe007-A, RRID: CVCL_C860, passages 45–80) derived at Masaryk University, Brno, and characterized in ref 55. hESCs were cultured at 37 °C with 5% CO_2_ and propagated on cell culture dishes coated with Matrigel (354277, hESC-qualified, Corning) in an hESC medium consisting of DMEM/F-12, 15% knockout serum replacement (10828–028, Gibco), 2 mM l-glutamine (25030–024, Gibco), nonessential amino acids (11140–035, Gibco), 1% penicillin/streptomycin (XCA4122/100, Biosera), 0.1 mM 2-beta-mercaptoethanol (M3148, Sigma-Aldrich), and 4 ng/mL human FGF2 (100–18B, Peprotech) conditioned by mouse embryonic fibroblasts (MEFs) with a fresh addition of 2 mM l-glutamine and 4 ng/mL hFGF-2.

Isolation of adipose-derived stromal cells (ADSCs) was performed in compliance with the ethical standards provided in the 1964 Declaration of Helsinki and its later amendments or comparable ethical standards and approved by the Ethics Committee St. Anne’s University Hospital Brno (8 V/2020). Written informed consent approving experimental use of extracted adipose tissue was obtained from the healthy donor before the liposuction procedure was applied. Adipose-derived stromal cells (ADSCs) were isolated by the centrifugation method from fat tissue, described in ref 56 and cryopreserved in passage 1. After thawing, ADSCs were expanded in DMEM + GlutaMAX (31966021, Gibco) and 10% FBS (FB-1101/500, Biosera) with the addition of 1% penicillin/streptomycin. Passages 3–9 were used for experiments.

Human umbilical vein endothelial cells (HUVECs, C2519A, Lonza) were grown in an Endothelial Cell Growth Medium 2 (EGM-2, CC3162, Lonza) with 1% penicillin/streptomycin. Passages 3–9 were used for experiments.

### 2.11 Formation of Spheroids

Agarose microwells were cast in silicone molds (3D Petri Dishes, Microtissues) following the manufacturer’s protocol. Briefly, 2% (w/v) agarose (A8963, PanReac Applichem) was dissolved in PBS and autoclaved. Molten agarose was poured into the silicone master mold and removed when solidified. Prepared agarose molds were seeded with cells in desired concentrations (200–800 cells/spheroid) and allowed to settle for 10 min. For the first 24 h, the medium was supplemented with 20 μM ROCK-inhibitor (Y-27632 2HCl, Selleckchem) to enhance aggregation of cells. Spheroids were harvested at day 2 or 3.

### 2.12 Evaluation of CaCl_2_ Concentrations in Cell Culture

The effect of CaCl_2_ on hESC growth was examined at concentrations of 0, 1, 2, 5, 10, 20, 50, and 100 mM in culture medium. hESCs were seeded on a Matrigel-coated surface at a concentration of 3.75 × 10^5^ cells/cm^2^, and 6 brightfield images were acquired 12, 24, and 48 h after cell seeding with the objective 10×/NA 0.3, DRY. The cell-covered area was measured manually for each field of view using ImageJ.

### 2.13 Evaluation of the Effect of Hydrogel Composition on Spheroid Fusion

Spheroid fusion was evaluated in agarose microwells designed to maintain spheroids in proximity during the experiments. To fabricate the microwells, 2% agarose was poured into 24 well-plate (24WP) wells, and a 3D printed custom-made stamp (Figure S2C) was inserted into the molten agarose. After solidification, the stamp was removed, creating a well with hemispherical microwells with a 500 μm × 500 μm base and a depth of 300 μm. Approximately 10 hESC spheroids were seeded into each stamped well and overlaid with 30 μL of alginate solution. After cross-linking, wells were filled with 1 mL of culture medium, with or without additional CaCl_2_. Fusion of spheroids was evaluated over 36 h. The number of individual objects and object area were used as metrics for quantification.

### 2.14 Evaluation of Cell Adhesion and Spreading on the Surface of Alginate Hydrogels

For cell adhesion assessment, 75 μL of alginate was spread onto a 24WP with a Ca­(OH)_2_-modified surface and polymerized with 100 mM CaCl_2_, first using the ultrasonic humidifier and later by gentle pipetting CaCl_2_ solution, to fully cover the gel for 30–60 s. Hydrogels were washed twice with DMEM/F-12, and cells were seeded onto their surfaces in concentrations of 3 × 10^5^ cells/cm^2^ (ADSCs, HUVECs) and 7.5 × 10^5^ cells/cm^2^ (hESCs). The culture medium was supplemented with 5 mM CaCl_2_.

### 2.15 Embedding Cells and 3D Culture in Hydrogels

Cells and spheroids, respectively, were immixed into the alginate solutions along with the medium during the dilution of stock polymer solutions. The cell-laden polymer solution was used for pipetting hemispherical domes or loaded into a cartridge for bioprinting (see further). Cell concentrations were related to specific cell types and experiments: 8 × 10^6^ cells/mL (hESCs; experiments with cross-linking temperature, Figure S2D), 4 × 10^6^ cells/mL (hESCs in TYRAY-8DS, Figure 4A), and 1 × 10^6^ cells/mL (ADSCs, Figure 4B). For formation of endothelial networks (Figure 4C), we combined 4.5 × 10^6^ cells/mL ADSCs with 1250 spheroids/mL HUVECs (400 cells per spheroid; corresponds to 0.5 × 10^6^ cells/mL). Both unmodified and TYRAY-8DS alginate in A2.5 and A1G10 formulations were used, along with Matrigel as a control.

Unmodified alginate A2.5 was pipetted onto Ca­(OH)_2_-modified polystyrene tissue culture plates, spread, and immediately aspirated from the bottom of the well. This thin bottom layer was polymerized with 100 mM CaCl_2_ using the ultrasonic humidifier to create a barrier between cells and the plastic. 40 μL of alginate solution with immixed cells was carefully pipetted on the alginate layer to form a dome-like shape and cross-linked with cold 100 mM CaCl_2_, first using the ultrasonic humidifier and then by gentle pipetting to fully submerge the dome, with gelation for 30–60 s. Hydrogels were quickly washed twice with DMEM/F-12 (RT) and subsequently cultured in a cell-specific medium containing 5–10 mM CaCl_2_. Medium was exchanged every 2–3 days.

For 3D cell culture in Matrigel, the hydrogel was thawed on ice and mixed with cell suspension at a 1:1 ratio on the parafilm surface. 40 μL of Matrigel domes was pipetted on the top of a thin layer of cell-free Matrigel to limit attachment of cells directly to the plastic surface. Domes were gelled for 30 min in the cell culture incubator before adding the cell culture medium.

### 2.16 3D Bioprinting

BioScaffolder 3.1 (Gesim) was used to print all structures, employing 3 mL of cartridges (Nordson) and dispensing needles (G22-ID = 0.41 mm, G25-ID = 0.25 mm, Nordson). The range of printing parameters included (i) a heating bed set to 37 °C for pure alginate boinks and 37 °C, 22 °C, and 4 °C for alginate-gelatin mixtures, (ii) speed from 10 to 50 mm/s, and (iii) extrusion pressure set between 30 and 50 kPa. Variation in these factors depended on bioink composition, needle size, and desired line thickness. The lowest pressure was selected to ensure stable extrusion. For design of 3D models of concentric circles and grid lines, Fusion360 (AutoCAD) and PrusaSlicer were used. To evaluate material printability, a printability index (Pr)^57^ was calculated from the shape of individual grid squares in the structure (Figure 2H) according to the formula

2 $$\mathrm{Pr}=\frac{{perimeter}^{2}}{16\times area}$$

Grid squares marked with yellow color in the schematic were selected for the analysis to assess the region that is the most representative and the least affected by the overall design geometry, i.e., created by a single straight pass of a printing nozzle at each side.

### 2.17 Fixation and Staining

Oct4 and E-cadherin expression in human embryonic stem cells printed in alginate hydrogels were assessed by immunofluorescent labeling. Cells were grown in printed hydrogels for 5 days and fixed with 4% paraformaldehyde dissolved in 0.1 M phosphate-buffered saline (PBS) for 20 min, followed by 3× PBS wash for 5 min and permeabilization by 0.1% Triton X-100 for 5 min. Samples were blocked with 5% BSA for 1 h and stained with primary antibodies against Oct-4 (2750, Cell Signaling) and E-Cadherin (3195, Cell Signaling) overnight at 4 °C. Constructs were washed 3 times with PBS and stained with secondary antibodies against mouse/rabbit AF488 and AF594 (A28175 and A150080, Invitrogen). Nuclei were counterstained with 1 μg/mL 4′,6-diamidino-2-phenylindole (DAPI, 32670, Sigma-Aldrich, Czech Republic). Images were acquired using a confocal laser scanning microscope, FluoView 500 (Olympus C&S Ltd., Prague, Czech Republic).

To evaluate the viability of cells, samples were stained with a live/dead fluorescent solution for 5 min at 22 °C. Fluorescent stock solution (1000×) contained 0.03% acridine orange (A6014, Sigma-Aldrich) and 0.1% ethidium bromide (46065, Sigma-Aldrich) in 2% ethanol. Live/dead cells assay was visualized using a confocal laser scanning microscope.

To visualize the endothelial networks, cells were stained by *Ulex europeaeus* agglutinin I conjugated with fluorescein (UEA, FL-1061, Vector laboratories). Gels were fixed with 4% formaldehyde and washed 3 × for 5 min, followed by incubation in blocking buffer for 1 h (1% BSA, 0.05% Tween 20 in PBS). Staining of endothelial cells was carried out using 10 μg/mL UEA in blocking buffer at RT for 2 h, and nuclei were counterstained with 1 μg/mL DAPI. Stained samples were washed in PBS with 0.05% Tween 20 (3 × 10 min) and imaged using a confocal laser scanning microscope (Zeiss LSM 700). When working with 3D alginate gels, all solutions were prepared in Tris-buffered saline with 5 mM CaCl_2_ instead of PBS.

### 2.18 Imaging, Image Processing, and Statistical Analysis

A motorized Olympus IX71 Cell̂R imaging station was used for live cells imaging with multiple fields of view acquired for each replicate. Image processing (calibration, timelapse sequence processing, and measurements) was conducted using ImageJ software.^58^ Parameters of endothelial networks (total network length, number of junctions) were measured using AngioTool.^59^ Network density was calculated as a ratio of total network length per image area. Data were analyzed using R ver. 4.0.5 ^60^ with packages ggplot ver. 3.3.3,^61^ rstatix ver. 0.7.0,^62^ and ggprism ver. 1.0.3.^63^ Pairwise comparisons were statistically analyzed using Wilcoxon test with Bonferroni correction to control false-positive error rate when using multiple tests. For all tests, significance levels were denoted as follows: * (*p* < 0.05), ** (*p* < 0.01), *** (*p* < 0.001), **** (*p* < 0.0001). All box-and-whisker plots represent median, extending from first to third quartile, with whiskers equal to 1.5 × interquartile range.

## 3 Results

### 3.1 Preparation and Cross-Linking of 3D Printed Alginate Hydrogels

Despite the significant progress made in alginate bioprinting, several challenges remain to be addressed to ensure the stable and reproducible creation of alginate constructs, which is essential for further cell observation and research. For instance, clear and homogeneous alginate solutions and hydrogels are crucial for reproducible cell culture, 3D bioprinting, and microscopy observations. To ensure consistent cell culture and 3D bioprinting outcomes, optimization of the bioink preparation and stock solution has to be performed. By employing a two-step process of dissolution, freeze-drying, and redissolution, we successfully produced clear, homogeneous, transparent, and lump-free alginate solutions suitable for bioink formulation (Figure S1A–C). Additionally, our method transformed crystalline alginate powder into a fibrous material, which could be quickly dissolved up to an 8% concentration. Lower concentration of the alginate during the initial dissolution (0.5%) allowed effective filtration, serving also as an effective sterilization method. We compared various sterilization methods, including filtration, UV irradiation, autoclaving, and gamma irradiation. Among these, 200 Gy gamma irradiation proved to be the most effective as it prevented recurrent bacterial growth observed with UV irradiation (Figure S1D,E) while preserving the flow characteristics of alginate solution (Figure S1F).

We investigated the impact of different CaCl_2_ application techniques on the cross-linking of alginate (Figures 1A,B and S1G). Microscopic analysis revealed distinct topographical changes and damage to alginate structures when pipetting and spraying CaCl_2_ solution. The size of imperfections caused by cross-linker droplets can reach up to a millimeter when using manual spraying and exceed this in the case of manual pipetting. In contrast, humidifier-produced aerosol creates only small craters generally in the range of 10–50 μm. This minimal surface irregularities are unlikely to interfere with 3D printed millimeter-scale structures or adhesion studies.

**Figure 1:**
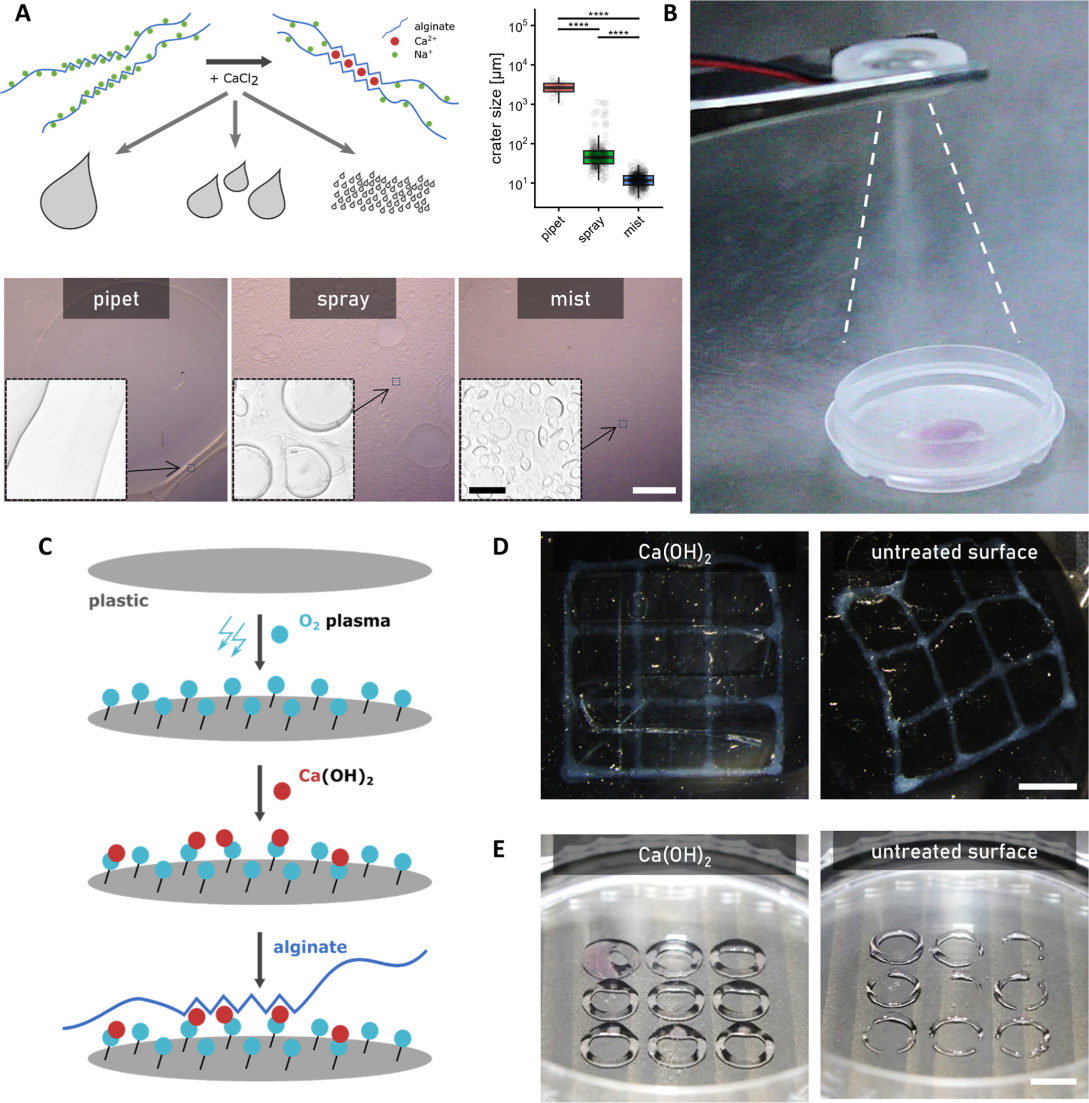
Optimizing alginate hydrogel cross-linking and anchoring for enhanced stability and precision. (A) CaCl_2_ application method influences the topography of alginate hydrogel surfaces. Scale bars 1 mm (overview, white) and 50 μm (insets, black). Data presented as Tukey-style boxplots with median, quartiles, whiskers = 1.5 × IQR, and outliers. *p < 0.05; **p < 0.01; ***p < 0.001; ****p < 0.0001. (B) Application of CaCl_2_ mist produced by an ultrasonic humidifier. (C) Surface modification of tissue culture plastic (TCP) surfaces with Ca­(OH)_2_ for stable anchoring of alginate hydrogels. Schematic illustrates oxygen plasma treatment and subsequent calcium immobilization on the TCP surface. (D) Stability comparison of alginate grid structures after 14 days in culture. Structures on untreated TCP detach and float, while those printed on Ca­(OH)_2_-modified surfaces remain securely anchored. Scale bar 5 mm. (E) Enhanced printing precision on the Ca­(OH)_2_-modified surface compared to that on untreated surfaces. Both structures were printed using identical alginate formulations and printing parameters. Scale bar 5 mm.

To maintain the stability of the printed alginate structures over extended periods, we supplemented the culture medium with additional CaCl_2_. Given that our experiments involved three distinctive cell types (hESCs, ADSCs, HUVECs), we optimized the calcium concentration using hESCs, the most sensitive of the three. No significant decrease in hESC growth was observed at concentrations up to 10 mM (Figure S1H,I). Supplementation with 5 mM CaCl_2_ was selected as optimal, where the alginate structures did not disintegrate during media exchange or handling. It also balanced the stability of the alginate constructs and minimized calcium precipitation, which became problematic at higher concentrations and volumes of medium (Figure S1J,K).

Cross-linked hydrogel structures often detached from culture plastics within a few hours, and 3D-printed alginate lines tended to tear and form droplets, particularly on untreated surfaces of polystyrene dishes. We addressed this issue by surface modification with Ca­(OH)_2_, providing crucial cross-linking of the alginate upon contact with deposited calcium ions, thereby enhancing the anchorage duration to over 14 days and facilitating reproducible and precise printing outcomes (Figures 1C–E and S1L).

## 4 Fine-Tuning the Alginate Bioink to Support Spheroid Fusion and Printability

In planar conditions, pluripotent stem cells typically form compact colonies. However, alginate matrices lack essential degradable sites, which limits cell migration, leading to cellular isolation and reduced survival. To overcome these limitations and facilitate the formation of interconnected tissue structures, we employed a strategy involving multicellular spheroids and providing cell–cell interactions, therefore promoting cell viability and proliferation, especially at the initial stages of culture. Multicellular spheroids, ranging from 100 to 300 μm in diameter, were formed in nonadherent agarose microwells and mixed into alginate solution for bioprinting (Figure 2A,B). The use of 22G nozzles (413 μm inner diameter) was shown to be large enough in diameter to facilitate the printing of spheroid-laden bioink without nozzle clogging or spheroid damage (Figure 2C). Notably, cells within printed spheroids exhibited significantly higher viability than those dispersed as single cells within the gel (Figure 2D). Immunolabeling confirmed the retention of pluripotency markers and intercellular junctions in printed hESC spheroids for at least 6 days (Figure 2E).

**Figure 2:**
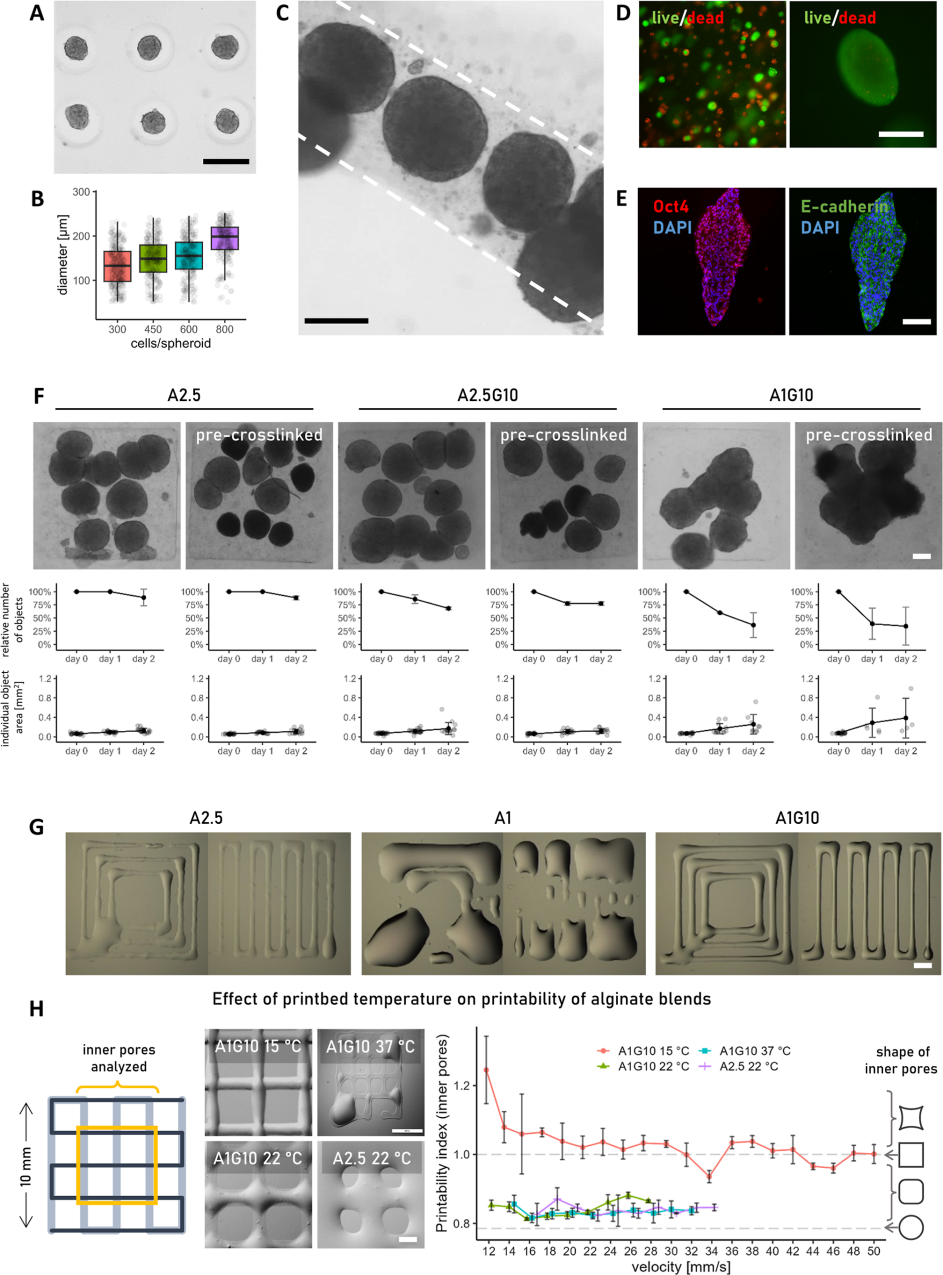
Fine-tuning the alginate bioink to support spheroid fusion and printability. (A) hESC spheroids are formed in nonadherent agarose microwells. Scale bar 200 μm. (B) Spheroid size is controlled by the number of seeded cells. Data presented as Tukey-style boxplots with median, quartiles, whiskers = 1.5 × IQR. (C) Spheroids printed in unmodified alginate retain their compactness during the printing process. Scale bar 200 μm. (D) After 6 days of culture, single cells embedded in alginate (4 × 10^6^/ml) often undergo cell death (red). Cells aggregated in spheroids were found to remain highly viable (green). Scale bar 200 μm. (E) hESCs were maintained pluripotent after printing. Spheroids often elongated during 3D culture in alginate. Scale bar 200 μm. (F) Fusion of hESC spheroids in different alginate hydrogels. The addition of gelatin supports the fusion only after the alginate concentration is reduced and works even with pre-cross-linked (10 mM CaCl2) alginate. Scale bar 200 μm. Data presented as mean ± SD (G) Enhanced printability of A1G10 compared to A2.5 and A1 alone. Scale bar 1 mm. (H) Printability of A1G10 is further improved via lowering the print bed temperature. A square grid was printed, and the structure of the inner pores marked by the yellow square was analyzed to evaluate the printability index. Scale bar 1 mm. Data presented as mean ± SD.

Due to the higher mass of spheroids, bioprinting posed challenges as they tended to sink to the bottom of the cartridge. To counter this, we used pre-cross-linked alginate, which has higher viscosity preventing spheroid sinking (Figure S2A).

While utilizing cell spheroids improved cell viability during long-term culture in 2.5% alginate hydrogels (A2.5), cell migration remained limited. Compared to Matrigel, almost every spheroid grew isolated within the alginate hydrogel matrix (Figures 2F and S2B). To enhance permeability and promote spheroid fusion, we explored strategies such as lowering alginate concentration and incorporating gelatin to create a loose alginate network. In order to evaluate spheroid fusion in alginate-gelatin bioink, we designed a 3D-printed stamp to cast patterned agarose wells (Figure S2C). That way, we could quickly and easily place spheroids in proximity in a variety of examined bioinks. While both pure A2.5 and A2.5 with additional gelatin (A2.5G10) supported spheroid growth, fusion of spheroids was rarely observed within 2 days (Figures 2F and S2B). Spheroid fusion was quantified by tracking the area and number of individual objects over time, where a sharp increase in area and a reduction in count indicated progressive fusion events. Reducing the alginate concentration to 1% in the mixed bioink (A1G10) led to a decreased number of individual spheroids and formation of substantially larger, fused structures, even in the pre-cross-linked variants with immixed CaCl_2_ (Figure 2F).

Moreover, we evaluated the printability of the alginate bioinks. The gelatin content in A1G10 facilitated the printing of otherwise unprintable A1 bioink (Figure 2G), which was too thin and usually resulted in separated droplets and broken lines when printed. The A1G10 blend exhibited significantly enhanced printability compared to A2.5, as evidenced by the printability index close to 1, which quantitatively assessed the individual grid squares within the printed structure (Figure 2H). Moreover, the printability range of A1G10 bioink on a cooled print bed at constant pressure was much wider as print speed could be set from 12 to 50 mm/s without losing the grid structure. Printing A2.5 on room temperature print bed and A1G10 on room temperature, or warmed, bed could be done only in the range of 16–34, 12–28, or 14–32 mm/s, respectively. In addition to affecting printing precision, cross-linking A1G10 bioink at lower temperatures enhanced cell growth, with small colonies emerging from single hESCs embedded in A1G10 when cross-linked at room temperature or on ice (Figure S2D). In contrast, when cross-linked at 37 °C or in A2.5, cell growth was significantly reduced.

## 5 Modification of Alginate with the TYRAY Peptide Allows Adhesion of Multiple Cell Types

To improve the adhesive properties of alginate hydrogels, we focused on evaluating the functionality of peptide modifications. Based upon our previous work with the TYRAY peptide,^49^ we extended the utilization of TYRAY alginate to 3D cell culture, cell types, and bioprinting technology. This study involved three distinct cell types (hESCs, ADSCs, HUVECs) to comprehensively assess their response to unmodified alginate, TYRAY-modified alginates with two peptide concentrations (expressed as degrees of substitution, DS), and a commercially available RGD-functionalized alginate (CELLINK A-RGD) selected as a widely used material. As the exact peptide substitution and coupling chemistry of the commercial A-RGD bioink are proprietary, this material was included as a functional benchmark rather than for quantitative peptide comparison. We first tested the adhesive properties by seeding cells on the top of a thin alginate layer, allowing reliable quantitative (cell adhesion) and qualitative (morphology) assessment of cell–material interaction. Among the alginates tested in this study, only TYRAY-modified alginate with high peptide amounts (8% DS, referred to as TYRAY-8DS) facilitated successful attachment and spreading of all three cell types (Figure 3A). Although the CELLINK A-RGD hydrogel partially promoted ADSC adhesion and spreading, its impact on HUVEC adhesion was limited, and no adhesion was observed with hESCs (Figure 3A). Successful adhesion was observed for ADSCs and HUVECs on gelatin-coated plastic and for hESCs on vitronectin-coated plastic; however, no adhesion was observed on unmodified alginate (Figure 3A). For ADSCs and HUVECs, the effect of higher peptide concentration in TYRAY-8DS became more pronounced after 72 h of culture, where a significant increase in the number of adhered cells was observed compared to TYRAY-1.8DS (Figure 3B).

**Figure 3:**
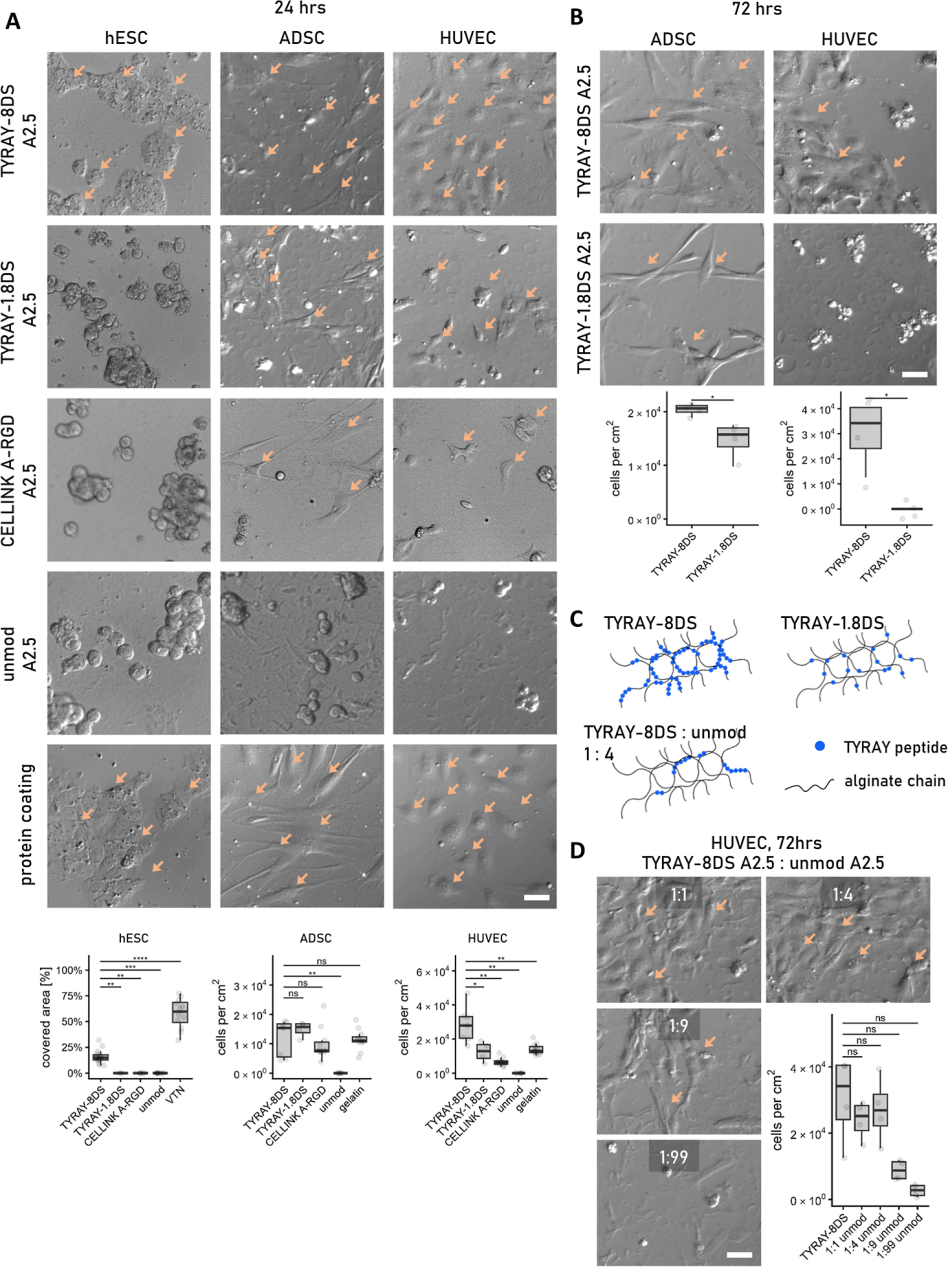
Enhanced cell adhesion, spreading, and growth on TYRAY-modified alginate hydrogels. (A) Comparative interaction of the three cell types seeded on the surface of TYRAY-modified alginate, commercially available CELLINK A-RGD, unmodified alginate hydrogels, and control surfaces (vitronectin-coated for hESCs, gelatin-coated for HUVECs, ADSCs) after 24 h of culture. (B) Increased peptide substitution TYRAY-8DS improves attachment and spreading of HUVECs and ADSCs after 72 h of culture. (C) Schematic depiction of peptide distribution in alginate hydrogels with varying degrees of peptide substitution, including blends of TYRAY-8DS with unmodified alginate. (D) Growth and spreading of HUVECs cultured for 72 h on TYRAY-8DS blended with unmodified A2.5 alginate. Scale bars 50 μm, examples of attached cells marked with arrows. Data presented as Tukey-style boxplots with median, quartiles, whiskers = 1.5 × IQR, and outliers. *p < 0.05; **p < 0.01; ***p < 0.001; ****p < 0.0001.

To assess whether the adhesive functionality of TYRAY-modified alginate is preserved upon volumetric dilution—a strategy applied in the A1G10 formulation—we next evaluated a mixture of TYRAY-8DS with unmodified alginate in ratios of 1:1, 1:4, 1:9, and 1:99. Comparison of the cell–material interaction of TYRAY-1.8DS and TYRAY-8DS mixed with unmodified alginate at 1:4 ratio with HUVECs revealed a clear difference in adhesion. Both compositions had similar overall peptide volume concentration (1.9 mM and 2.2 mM respectively), but the key difference was in locally heterogeneous peptide distribution with possible formation of peptide rich microdomains (Figure 3C), which we believe influenced the observed cellular responses. Remarkably, both HUVECs and ADSCs adhered and spread well even at 1:9 dilution (Figures 3D and S3A), with ADSCs showing slight proliferation (Figure S3B). At 1:99 dilution, only limited adhesion was observed without proliferation (Figures 3D and S3A,B). Importantly, these adhesive properties were preserved following gamma sterilization (Figure S3C), confirming the robustness of the peptide-functionalized matrix.

## 6 TYRAY A1G10 Formulation Supports Cell Expansion and Formation of Capillary Networks in 3D

Our investigation demonstrates a significant improvement in the survival and proliferation of single hESCs when cultured within 3D TYRAY-8DS alginate hydrogels, as compared to both unmodified alginate and CELLINK A-RGD (Figure 4A,B). While the addition of gelatin enhanced growth of hESCs in unmodified alginate, this improvement was not observed when gelatin was incorporated into CELLINK RGD alginate (Figure 4C). The A1G10 formulation of TYRAY-8DS resulted in slightly decreased colony diameter (Figure 4C). Importantly, both A2.5 and A1G10 TYRAY alginates showed a high fraction of living, proliferative, colony-forming cells (Figure 4D), with a minority of cells that did not grow during the 4 days as these were considered dead and excluded from the analyses. Among the tested materials, only Matrigel surpassed TYRAY-8DS in supporting hESC growth.

**Figure 4:**
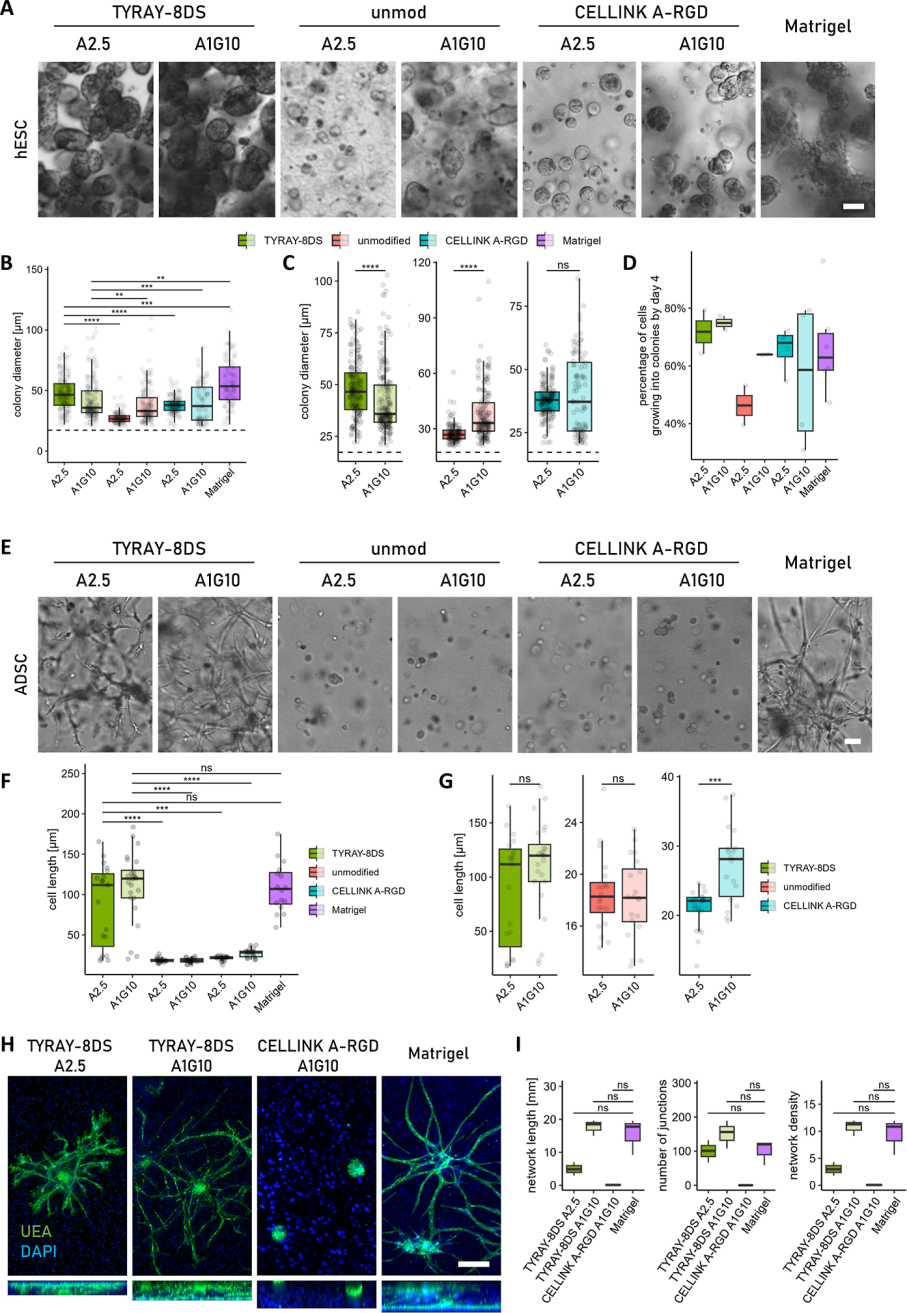
TYRAY-modified alginates support cell growth, migration, and formation of networks within the hydrogel. (A) hESCs formed spherical 3D colonies within 4 days in supporting hydrogels. Scale bar 50 μm. (B) Quantification of colony size across tested hydrogels. The dashed line indicates the average diameter of single hESCs immediately after embedding; objects below this threshold (presumed nonviable cells) were excluded from analysis. (C) Gelatin addition improved size of colonies in unmodified alginate but had no positive effect in TYRAY-modified alginate or CELLINK A-RGD. The dashed line again denotes the average diameter of single cells right after embedding. Nonviable cells were excluded. (D) The percentage of cells that successfully proliferated and formed colonies was consistently high in both TYRAY alginates. (E) ADSCs exhibited pronounced spreading and migration in TYRAY alginate but not in CELLINK A-RGD or unmodified alginate after 7 days of culture. Scale bar 50 μm. (F) TYRAY alginate supported ADSCs elongation and protrusion formation in a manner comparable to that in Matrigel, unlike unmodified or CELLINK A-RGD alginates. (G) In the presence of stromal cells, gelatin appeared to modestly enhance cellular spreading. Measurements represent the length (diameter if round) of clearly identifiable individual cells. (H) HUVEC aggregates cocultured with stromal cells formed endothelial networks in different hydrogels during 14 days (maximum projections in XY and YZ). TYRAY alginate supported limited endothelial spreading (green), which was further enhanced by gelatin and allowed formation of a branched network. No sprouting was observed in CELLINK A-RGD. Matrigel was used as a positive control. Scale bars 100 μm. (I) Quantification of endothelial networks in hydrogels shows increased network length, number of junctions, and network density in TYRAY A1G10 as opposed to CELLINK A-RGD. Network density was calculated as a ratio of total network length per area. *n* = 3. Data presented as Tukey-style boxplots with median, quartiles, whiskers = 1.5 × IQR, and outliers. *p < 0.05; **p < 0.01; ***p < 0.001; ****p < 0.0001.

ADSCs similarly exhibited extensive spreading, migration, and cell–cell interaction when cultured in TYRAY-8DS hydrogels for 7 days (Figure 4E,F). The presence of TYRAY had a pronounced effect as virtually we did not see any spreading in neither unmodified nor CELLINK A-RGD alginate (Figure 4E,F). The addition of gelatin had minimal impact on ADSCs spreading across most formulations, except for the CELLINK A-RGD, where a modest improvement was noticed (Figure 4G). However, even in the A1G10 formulation, cell morphology was limited to a slight elongation, in comparison to the pronounced protrusions observed in TYRAY alginate or Matrigel. This enhanced cellular behavior can be attributed to the TYRAY peptide’s role in promoting cell adhesion and interaction, which are essential for processes like differentiation and tissue formation.

The potential of TYRAY-modified alginates for tissue engineering applications was further supported by its ability to facilitate the formation of endothelial networks within the hydrogel. When human umbilical vein endothelial cells (HUVECs) were cocultured with stromal cells in the TYRAY-modified alginates, they formed short endothelial branches in TYRAY A2.5 hydrogels, indicating initial stages of network formation. In contrast, looser TYRAY A1G10 hydrogels enabled development of longer and more interconnected endothelial networks, with the network length and density comparable to that in Matrigel (Figure 4H,I). Such a level of network organization is a crucial milestone toward engineering functional, vascularized tissue constructs. By comparison, neither unmodified alginate (data not shown) nor CELLINK A-RGD supported appreciable endothelial migration or network formation (Figure 4H), highlighting the limitations of conventional bioinks in promoting complex multicellular behavior. The ability of TYRAY A1G10 in promoting robust endothelial organization underscores its promise for applications in vascular tissue engineering, including the development of prevascularized scaffolds designed to enhance implant integration and long-term viability in vivo.

Overall, our findings demonstrate that TYRAY-modified alginates, particularly the A1G10 formulation, provide a highly supportive microenvironment for the culture and expansion of various cell types, including hESCs, ADSCs, and endothelial cells. Beyond enhancing cell viability and proliferation, these hydrogels enable complex multicellular behaviors such as spheroid fusion, cell migration, and the formation of endothelial networks. This multifunctionality positions TYRAY-modified alginates as a promising platform for advanced applications, including spatially organized cocultures, engineered microtissues, and studies of cell interactions with the microenvironment. Finally, the results underscore the versatility of this material system for developing functional, bioprinted tissue constructs, addressing persistent challenges in the field.

## 7 Discussion

In 3D cell culture and bioprinting applications, creating biocompatible, stable structures that support cell adhesion, proliferation, and vascular network formation remains challenging. Bioinks must provide support for both cell functionality and structural integrity in the printed constructs, particularly for delicate cell types like human pluripotent stem cells and endothelial cells.^16,64^

Traditionally utilized alginate hydrogels are often unstable in long-term culture and lack the sites for cell adhesion, which are crucial for effective cell matrix interactions. In this study, we resolve these challenges by modifying alginate with the TYRAY peptide combined with strategic protocol adjustments to generate an optimized bioink highly suitable for 3D bioprinting. By introducing integrin-binding peptides, fine-tuning cross-linking strategies, and utilizing multicellular spheroids, our approach significantly improves upon conventional methods, creating a stable, biomimetic environment that enhances stem cell survival, proliferation, and self-assembling, endothelial network formation, and structural stability in 3D tissue constructs that usually involve multiple cell types.

## 8 Improvements in Manipulation, Bioprinting of Alginate, and 3D Cell Culture

Achieving homogeneity and stable cross-linking in 3D alginate hydrogels is essential as it significantly impacts migration, survival, and/or differentiation.^65–67^ Ensuring optical transparency and sterility is essential for effective 3D cell culture and microscopic analysis. While purification steps, such as dialysis, are necessary for chemically modified alginates,^68^ they are typically not included in protocols for preparing unmodified alginate hydrogels. Filtration of low-concentration alginate has been proposed as an effective sterilization method, offering an alternative to autoclaving, high-dose gamma irradiation, or UV exposure.^69,70^ In this study, we demonstrate that a two-step preparation process—comprising lyophilization with dissolution followed by filtration—can be successfully applied even to unmodified alginates that did not undergo dialysis. This approach yields homogeneous, optically clear, and cost-effective hydrogels that are comparable in quality to purified commercial purified alternatives. Furthermore, while conventional rheological measurements (*G*′/*G*″, temperature sweeps) are valuable, our objective here was to establish a simple comparative tool for routine laboratory preparation of alginate bioinks. The droplet sliding assay fulfilled this role, enabling reproducible comparison of flow characteristics across formulations in a manner directly linked to practical bioprinting performance.

For sterilization of healthcare-related materials, high-dose gamma irradiation (15 and 25 kGy) is recommended according to ISO 11137–2:2013;^71^ however ,such doses can compromise the structural integrity of alginate.^72,73^ In contrast, our findings demonstrate that low-dose gamma irradiation at 200 Gy effectively eliminates microbial contamination without affecting mechanical properties of alginate, making it a practical and efficient sterilization method for routine in vitro applications. While we acknowledge that such low dosage may not eliminate heavy contamination, it proved effective as a functional step for preparation of bioinks and cell culture material in a clean, though not strictly sterile, laboratory environment.

Hydrogel stability is also influenced by the method of cross-linking. Traditional approaches, such as pipetting or spraying CaCl_2_ solution, often produce an uneven surface and disrupt delicate structures. While gentle support bath-based approaches can prevent deformation during cross-linking,^74^ they are less suitable for creating smooth, flat surfaces, and their preparation can be time-consuming. To overcome these limitations, we employed a hand-held ultrasonic humidifier to controllably deliver CaCl_2_ aerosol, which enabled uniform cross-linking while preserving surface topology. Compared to previously published methods using higher CaCl_2_ concentrations,^75^ we achieved effective cross-linking with a lower CaCl_2_ concentrations (100 mM), allowing for improved reproducibility and minimal impact on cell behavior.

A common issue in 3D bioprinting with alginate is the difficulty of anchoring constructs to culture surfaces as constructs tend to detach during extended culture or medium exchanges. This limits their utility, fidelity, and suitability for microscopy observation in long-term studies. One approach to address this issue is increasing the surface charge of culture dishes using poly lysine to strengthen electrostatic interactions with alginate. While this method is straightforward, poly lysine and other polycations have been reported to cause cytotoxicity and/or altered cellular behavior.^76,77^ We therefore invented and applied a surface modification with Ca­(OH)_2_, which provided strong and stable fixation of the alginate constructs to cell culture plastic for over 14 days, even with frequent medium changes. By enhancing scaffold adherence, we were able to support long-term cell culture without structural displacement, which addresses a critical gap in bioprinting and provides a reproducible method for securing alginate constructs, especially when working with sensitive cell types like hPSCs. Notably, we did not observe any adverse effects on cell morphology or viability when culturing on Ca­(OH)_2_-treated plastic.

Additionally, to counteract the leaching of calcium ions and subsequent gel disintegration,^78,79^ we supplemented the culture medium with 5 mM CaCl_2_. This concentration helped maintain the construct’s stability over extended periods, without causing precipitation that compromised optical transparency or introducing additional variability through cyclic changes in mechanical properties associated with recross-linking.^80^ Importantly, this supplementation did not adversely affect the growth of hESCs, nor ADSCs and HUVECs, supporting its suitability for diverse cell types in long-term culture.^81–83^

The inert nature of alginate bioinks presents a significant limitation, restricting cell migration and leading to isolated cell growth.^84^ This is particularly problematic in 3D bioprinting, where effective cell–cell interactions are crucial for biomimicking the tissue architecture and function, especially in stem cell and endothelial cultures.^21^ To overcome this challenge, we incorporated multicellular spheroids into the alginate solution prior to bioprinting. This strategy preserved essential cell–cell interactions, which promoted cell viability and proliferation more effectively than single-cell dispersions. Notably, hESC spheroids maintained their pluripotency markers and intercellular junctions within the printed constructs, enabling sustained growth and functionality over extended periods. To systematically evaluate spheroid fusion across different bioink formulations, we developed a custom 3D-printed stamping tool to generate patterned agarose microwells. While spheroid fusion has been described in the literature, standardized methods for assessing this process in hydrogel environments remain limited. Our setup enables reproducible spatial arrangement of spheroids and allows quantification of fusion dynamics via simple image-based analysis. This accessible platform offers a practical approach for studying how material properties influence tissue self-organization.

Embedding spheroids in bioinks aligns with recent research advocating for multicellular aggregates as a strategy to enhance cell survival and function in 3D cultures.^6,85,86^ Unlike single cells, which often fail to survive due to insufficient ECM interactions, spheroids provide intrinsic cell signaling and support in vivo-like environments, thereby supporting more physiologically relevant tissue models.

## 9 TYRAY Peptide Modification for Enhanced Cell Adhesion and Network Formation

As alginate lacks cell-adhesive properties, which limits cell attachment, growth, and also formation of vascular-like networks, its functionalization with biomimetic peptides has been broadly utilized.^87,88^ Here, we show that the inclusion of TYRAY-modified alginate provides beneficial enhancement of material properties, offering significant improvements in cell adhesion, particularly for hESCs, but also ADSCs and HUVECs. Functionality of the TYRAY peptide primarily for hESC adhesion was verified under 2D conditions,^35^ on modified surfaces of tissue culture plastic, and our previous work showed adhesion and spreading of hESCs on TYRAY-modified alginate surfaces.^49^ We carried out a set of experiments under planar conditions also in this work. As opposed to embedding cells in 3D hydrogels, 2D conditions provide a clear readout and allow reliable assessment of cell–material interaction through analysis of adhesion, spreading, cell morphology, and proliferation. Valuable insight gathered in this initial phase was then applied in subsequent 3D cultures.

Here, we demonstrate that higher functionalization of alginate with TYRAY is necessary for optimal hESC and HUVEC adhesion and proliferation, whereas ADSCs responded well also to lower concentrations of peptides. TYRAY-modified alginate demonstrated enhanced support for pluripotent and endothelial cell adhesion and organization compared to conventional RGD-modified bioinks under the tested conditions. We showed that heterogeneous distribution of adhesive motifs within the alginate matrix, rather than a uniform distribution or overall concentration, enhanced cell–material interactions. This finding aligns with earlier studies emphasizing the role of spatial organization as well as ligand availability in improving bioactive material efficacy,^89–91^ while the reduced overall concentration of the peptide offers economic benefits for research and potential applications in regenerative medicine.

Our findings also reveal that embedding hESCs and ADSCs within TYRAY-modified alginate hydrogels significantly improves cell proliferation, elongation, and migration. This cellular behavior closely resembles that observed in Matrigel, commonly used as an extracellular matrix surrogate. In the context of hESCs, TYRAY modification delivers essential bioactive cues that support not only cell survival but also sustained expansion. To further elucidate the influence of TYRAY peptides on differentiation pathways, future studies employing specific differentiation markers will be necessary. Given the known multipotency of ADSCs and their relevance in regenerative medicine, their ability to spread within TYRAY-modified hydrogels is particularly encouraging. This represents a marked improvement over predominantly round or oval cell morphology reported by others^32,92,93^ and observed in commercial RGD-alginate in the present work. Therefore, the bioactive microenvironment created by TYRAY-functionalized alginate could be used to direct cell differentiation toward target tissue types.

Finally, the enhancement was particularly pronounced with endothelial cells, where TYRAY-modified alginate facilitated the formation of well-organized, branched endothelial networks. By supporting integrin-mediated interactions tailored to hPSCs and endothelial cells,^43,44^ TYRAY-modified alginate offers a robust and versatile bioink that addresses the limitations of conventional alginates. Recently, bioprinting of vascularized constructs often utilizes gelMA or gelatin.^94–96^ However, the animal origin of these materials restricts their usage in regenerative tissue engineering and hPSC culture, where defined and xeno-free conditions are preferred. With synthetically modified hydrogels, the formation of vascular networks in synthetically modified hydrogels has mainly focused on the use of adhesive peptides RGD, YIGSR, IKVAV, REDV, and MMP-cleavable linkers, or combinations thereof.^97–100^ Their effectiveness is usually reported for hydrogels implanted in vivo, where several biological processes are involved, e.g., inflammation, hypoxia, and graft remodeling with the participation of many cell types. In vitro, formation of vascular networks has been described in various hydrogels, including fibrin, collagen, gelatin, Matrigel, or synthetic gels such as PEG, as reviewed in refs 101–104 . To our knowledge, self-organization of stable endothelial networks in alginate was before achieved only by seeding cells within premade alginate channels and tubes^105^ or a dried porous alginate scaffold.^106^ Within a bulk alginate hydrogel, the formation of interconnected networks was observed only rarely, without the characteristic capillary-like branched appearance.^107^ Our results therefore represent a critical advancement for the creation of vascularized tissue constructs, essential for engineering large-scale, functional tissues.

## 10 Future Directions and Implications

While TYRAY-modified alginate presents substantial advancements in supporting 3D cell culture and bioprinting applications, several areas require further exploration. First, high peptide concentrations, though effective, could impact scalability and cost-effectiveness, especially in larger-scale tissue models. Strategies to optimize peptide distribution or introduce mixed bioinks may allow for a more economical approach without sacrificing efficacy. Furthermore, while the ionic cross-linking of alginate provides initial stability, alternative cross-linking methods, such as covalent or enzymatic cross-linking, could enhance the long-term stability of constructs, particularly in dynamic environments. Moreover, combining two cross-linking methods could enable the use of alginate as a microgel bioink,^108^ an approach that holds promise for bioprinting vascularized tissue models.^95^ Furthermore, alginate represents a conservative baseline for bioink development due to its minimal intrinsic bioactivity and weak mechanical support compared to protein-based matrices such as GelMA, collagen, or fibrin. Demonstrating significant improvements in this context suggests that TYRAY functionalization and complementary strategies such as controlled cross-linking and hydrogel anchoring are likely to be transferrable to other hydrogel systems with greater inherent bioactivity and structural support. While we did not experimentally test GelMA or hyaluronic acid in this work, these represent promising directions for future validation.

In the context of hPSCs, our study focused on demonstrating survival, proliferation, and colony formation within TYRAY-modified alginate. We did not extend our experiments to long-term maintenance of pluripotency or directed differentiation as these require additional protocols and extended timelines. Future work will address these aspects to further establish the bioink’s utility in guiding lineage-specific outcomes. Such studies would provide complementary insights into how TYRAY-modified alginates support not only cellular survival and growth but also differentiation, a critical factor for translational applications in regenerative medicine.

## 11 Conclusion

In this study, we demonstrate the efficacy of TYRAY-modified alginate as a bioink for overcoming critical challenges in 3D bioprinting, offering significant improvements in cell adhesion, structural stability, and vascular network formation. By strategically modifying the alginate matrix with integrin-binding peptides and employing newly reported advanced cross-linking methods, we provide a biomimetic hydrogel that closely mimics the physiological environment, supporting the complex behaviors of stem and endothelial cells. These advances display TYRAY-alginate as a promising material for applications in regenerative medicine, particularly in the development of vascularized, tissue-specific constructs essential for translational therapies. Future research should aim at scaling and further optimization of these bioinks, potentially enhancing their application in clinical and industrial tissue engineering and broadening the scope of 3D bioprinting utilization in medicine and research.

## Supplementary Material



## Data Availability

The data that support the findings of this study are available upon reasonable request from the authors.

## Notes

Preparation, sterilization, and cross-linking of alginate hydrogels (Figure S1); characterization and growth of hESCs spheroids (Figure S2); ADSC response to TYRAY-modified alginate dilution and gamma sterilization (Figure S3); and ^1^H NMR spectra for alginate compounds (Figure S4) (PDF)
