# Supplementary material for: TYRAY-Functionalized Alginate Bioinks for 3D Bioprinting Support Stem Cell Culture and Endothelial Network Formation
Source: ACS Biomater Sci Eng. 2025 Nov 20;11(12):7368–83. doi: 10.1021/acsbiomaterials.5c01132 (PMC12690512; doi:10.1021/acsbiomaterials.5c01132)
Supplement: Supplementary file 1 [file ab5c01132_si_001.pdf]

SUPPLEMENTARY FIGURES

to

TYRAY-functionalized alginate bioinks for 3D bioprinting support stem cell culture and endothelial network formation

Chochola Vaclav[1], Spustova Karolina[1,7], Lavicky Josef[1], Golunova Anna[3], Pospisil Jakub[1], Dvořáková Jana[3], Kotelnikov Ilya[3], Kandra Mario[1], Streit Libor[4,5], Szklanny Ariel[6], Levenberg Shulamit[6], Proks Vladimir[3], Hampl Ales[1,2], Jaros Josef[1,2,*]

[1] Department of Histology and Embryology, Faculty of Medicine, Masaryk University, Kamenice 5, Brno, 62500, Czech Republic

[2] International Clinical Research Center, St. Anne's University Hospital Brno, Pekarska 53, Brno, 656 91, Czech Republic

[3] Institute of Macromolecular Chemistry, Czech Academy of Sciences, Heyrovskeho nam. 2, 16200 Prague, Czech Republic

[4] Department of Burns and Plastic Surgery, University Hospital Brno & Faculty of Medicine, Masaryk University, Jihlavska 20, Brno, 625 00, Czech Republic

[5] Department of Plastic and Aesthetic Surgery, St. Anne's University Hospital & Faculty of Medicine, Masaryk University, Pekarska 53, Brno, 656 91, Czech Republic

[6] Department of Biomedical Engineering, Technion – Israel Institute of Technology, Haifa, 32000 Israel

[7] Department of Molecular Cell Biology, Institute for Cancer Research, Oslo University Hospital, 0379 Oslo, Norway

[*] Corresponding author: jaros.josef@hotmail.com

Content

Figure S1: Preparation, sterilization, and crosslinking of alginate hydrogels.

Figure S2: Preparation, characterization and growth of hESCs spheroids in alginate hydrogels.

Figure S3: ADSC response to TYRAY-modified alginate dilution and gamma sterilization.

Figure S4: ^{1}H NMR spectra of alginate and TYRAY-modified alginates.

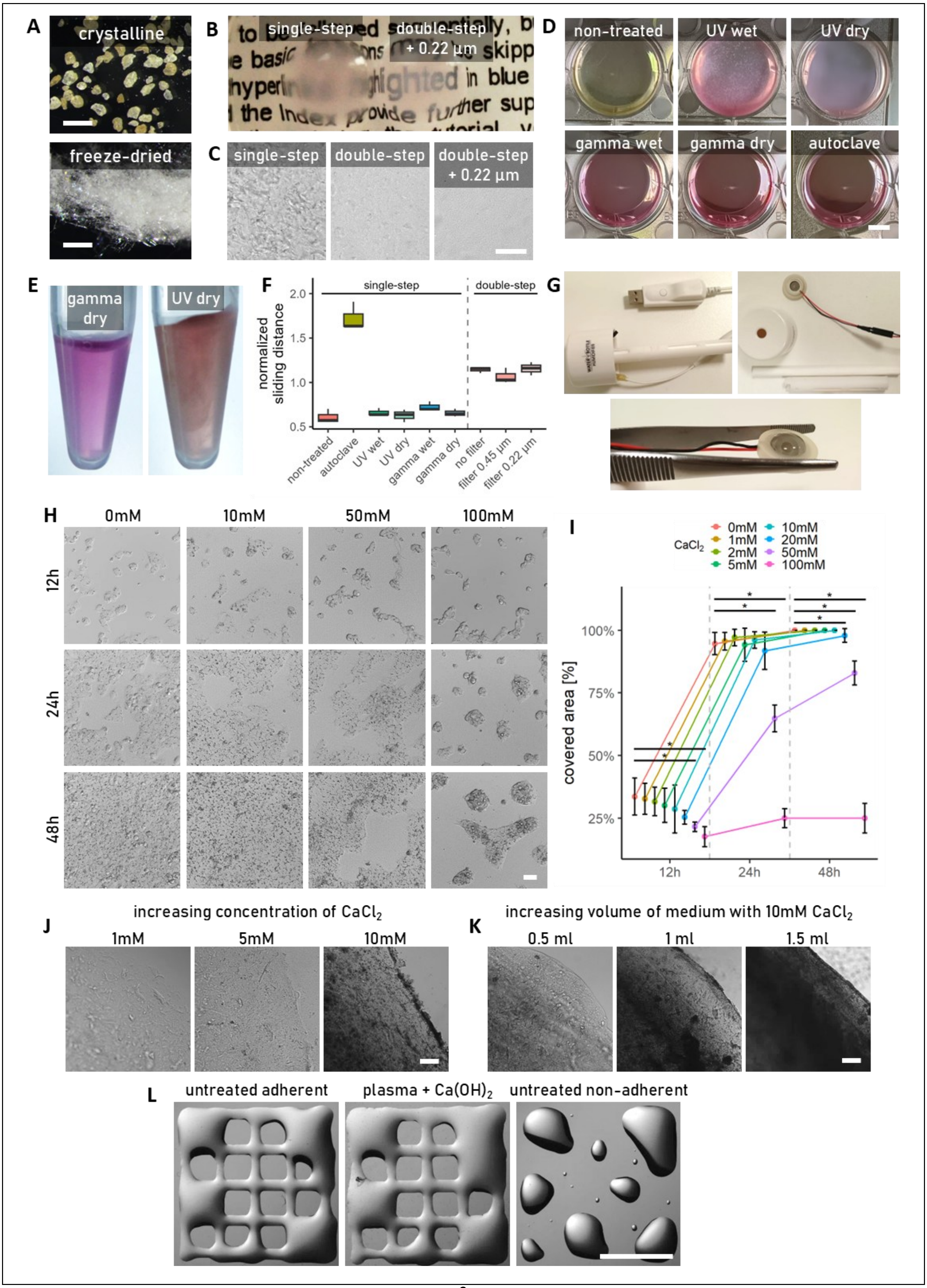
A
crystalline
freeze-dried
B
single-step
double-step
+ 0.22 µm
C
single-step
double-step
double-step
+ 0.22 µm
D
non-treated
UV wet
UV dry
gamma wet
gamma dry
autoclave
E
gamma
dry
UV dry
F
single-step
double-step
normalized
sliding distance
2.0
1.5
1.0
0.5
non-treated
autoclave
UV wet
UV dry
gamma wet
gamma dry
no filter
filter 0.45 µm
filter 0.22 µm
G
H
0mM
10mM
50mM
100mM
12h
24h
48h
I
CaCl2
0mM
1mM
2mM
5mM
10mM
20mM
50mM
100mM
covered area [%]
100%
75%
50%
25%
12h
24h
48h
*
J
increasing concentration of CaCl2
1mM
5mM
10mM
K
increasing volume of medium with 10mM CaCl2
0.5 ml
1 ml
1.5 ml
L
untreated adherent
plasma + Ca(OH)2
untreated non-adherent

Figure S1: Preparation, sterilization, and crosslinking of alginate hydrogels. ***A***. *Difference between dry alginates used for the stock solution preparations. The commercially available crystalline form of sodium alginate (top) and freeze-dried product (bottom). Scalebar 1 mm.* ***B***. *Overall transparency of crosslinked A2.5 domes from alginate domes prepared by the single-step dissolution, and double-step method - dissolution, lyophilization, and re-dissolution.* ***C.*** *Microscopy images showing undissolved speckles in alginate prepared by a single-step, and higher optical clarity of 50 µl alginate domes prepared by a double-step preparation method. Uncrosslinked, scalebar 50 µm.* ***D.*** *Cell culture medium was incubated with 100 µl of alginate and visually inspected. Alginate solutions were prepared or treated using different methods and stored at 4 °C for two weeks before testing. Contamination becomes apparent within 7 days of culture as change in color and/or visible microbial growth (turbidity, mold). Scalebar 5 mm.* ***E.*** *Successfully sterilized (gamma dry) and contaminated (UV dry) stock 5% alginate two weeks in the fridge. Contaminated alginate usually changes colour from pink to red, orange or yellow and becomes visibly turbid.* ***F.*** *Graph showing variations in flow behavior of 5% alginates prepared in water upon different types of sterilization/preparation, compared by sliding distance of alginate droplet deposited on a glass slide. All samples were normalized to the sliding distance of glycerol. N = 3. Data presented as Tukey-style boxplots with median, interquartile range and whiskers = 1.5×IQR.* ***G.*** *The transducer from USB ultrasonic humidifier was removed from the plastic casing, held with tweezers and used to gently apply $CaCl_2$ mist onto the alginate.* ***H.*** *Representative images of hESC growth under planar conditions, with elevated concentrations of $CaCl_2$. Scalebars 50 µm.* ***I.*** *Quantification of hESC growth in different concentrations of $CaCl_2$. N = 3, *$p < 0.05$, data presented as mean ± SD.* ***J.*** *Accumulation of insoluble precipitate (dark spots, areas) after incubation of alginate hydrogel in media with different concentrations of $CaCl_2$ for 13 days. Images show the edge of the hydrogel. Scalebars 50 µm.* ***K.*** *Accumulation of insoluble precipitate after incubation of alginate hydrogel in different volumes of medium with the same concentration of 10 mM $CaCl_2$ for 13 days. Images show the edge of the alginate. Scalebars 50 µm.* ***L.*** *Enhanced printing precision on modified surface. With the help of plasma treatment, Ca^{2+} can be utilized to print alginate on non-adherent tissue culture plastic. Scalebar 5 mm.*

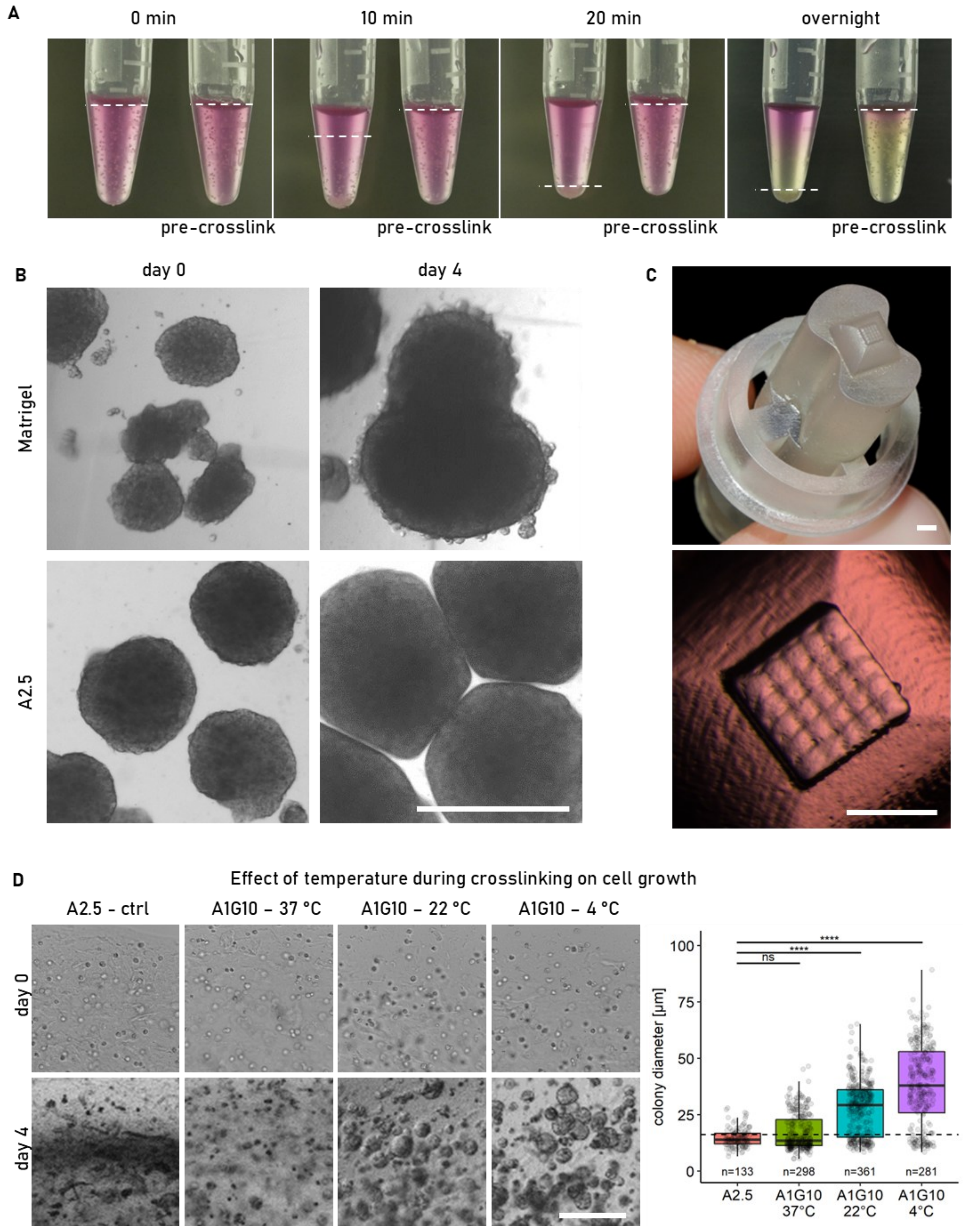

Figure S2: Preparation, characterization and growth of hESCs spheroids in alginate hydrogels. *A. Pre-crosslinking of alginates with 10mM* $CaCl_2$ *prevents cell spheroids from sinking in the tube/printing cartridge. Dashed line indicates position of topmost aggregates.* ***B.*** *In Matrigel, hESC spheroids fuse freely, but in A2.5 they*

grow separated. Scalebar 200 µm. ***C.*** *3D-printed stamp and a detail of the produced agarose well for evaluation of spheroid fusion in hydrogels. Scalebar 1 mm.* ***D.*** *Growth of cell aggregates formed from single cells after deposition and crosslinking of alginate-gelatin bioink on a bed with controlled temperature (37, 22 or 4 °C). Scalebar 50 µm. The graph represents the size of the colonies cultured over 4 days culture. Dashed line in the graph represents the mean diameter of seeded single cells right afterembedding. Data presented as Tukey-style boxplots with median, quartiles, whiskers = 1.5×IQR and outliers. *p < 0.05; **p < 0.01; ***p < 0.001; ****p < 0.0001.*

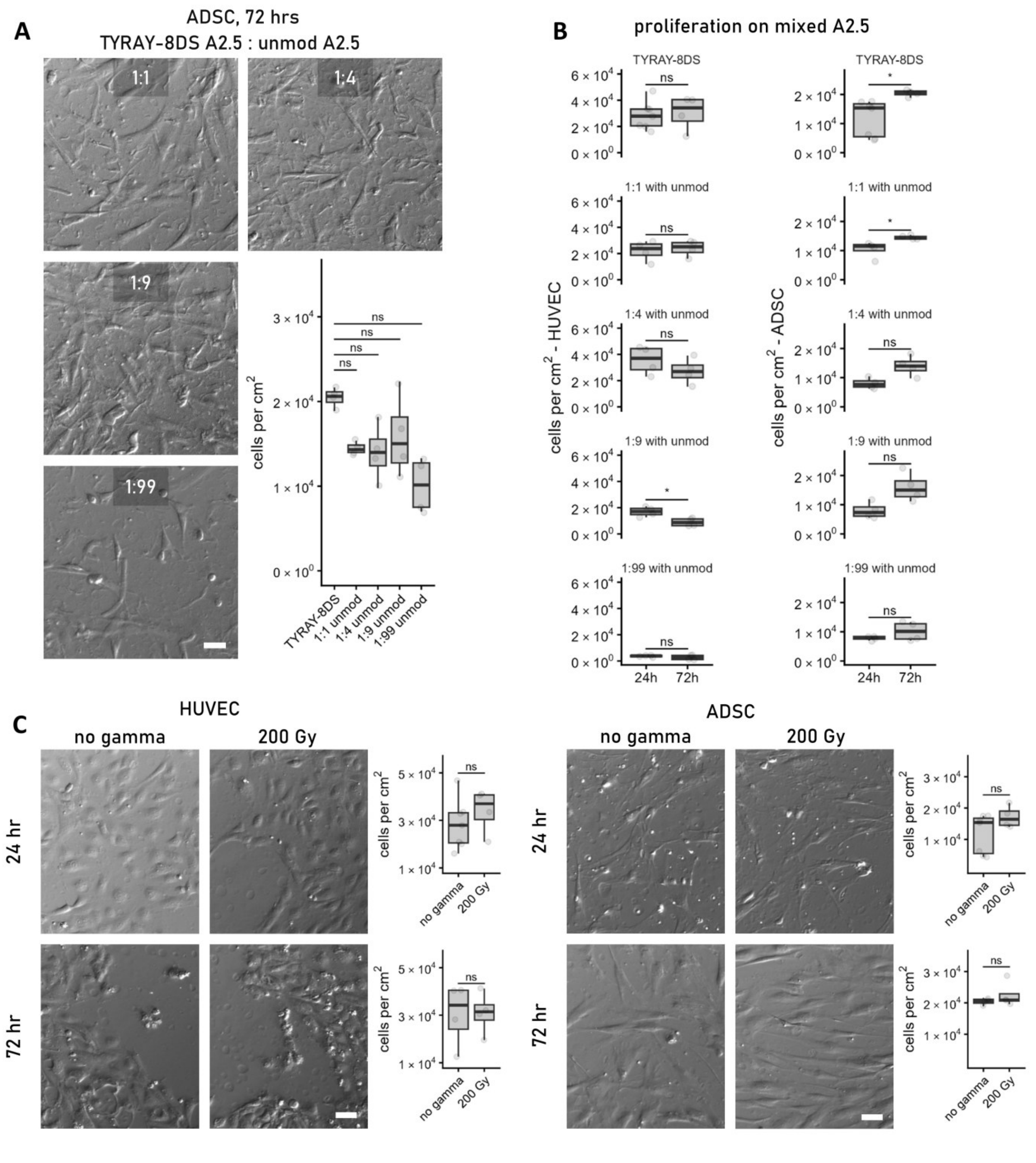

Figure S3: ADSC response to TYRAY-modified alginate dilution and gamma sterilization. ***A.*** *ADSC growth during 72 hrs on TYRAY-8DS mixed with unmodified A2.5. Positive effect of high peptide content is visible even at 1:9 ratio.* ***B.*** *Assessing proliferation of HUVECs and DPSCs on TYRAY-8DS mixed with unmodified alginate.* ***C.*** *Functionality of RGDT modified alginates (TYRAY-8DS A2.5) was preserved after gamma sterilization. Scalebars 50 µm. Data presented as Tukey-style boxplots with median, quartiles, whiskers = 1.5×IQR and outliers.* *$*p < 0.05$; $**p < 0.01$; $***p < 0.001$; $****p < 0.0001$.*

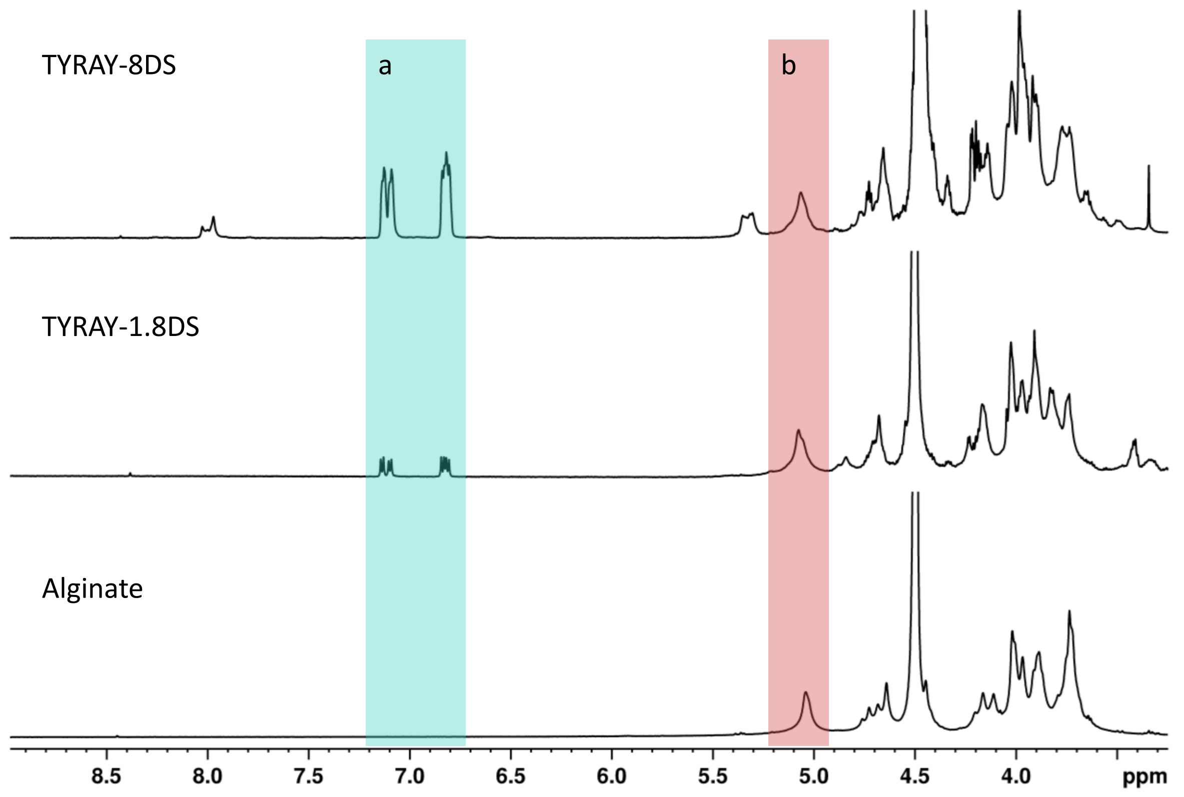

Figure S4: ¹H NMR spectra of alginate and TYRAY-modified alginates. *Representative spectra of unmodified alginate (bottom), TYRAY-1.8DS (middle), and TYRAY-8DS (top) were measured in* D_2O *at 50 °C. Hydrogens assigned to tyramine in the TYRAY peptide (a) and the C1 of guluronic acid in alginate (b) are highlighted in color, confirming successful peptide conjugation at different degrees of substitution.*
